# Exploring ammonium salt doping to enhance ion abundance for quantitative mass spectrometry imaging

**DOI:** 10.1007/s00216-025-06198-z

**Published:** 2025-11-04

**Authors:** Alora R. Dunnavant, Seth M. Eisenberg, David C. Muddiman

**Affiliations:** Biological Imaging Laboratory for Disease and Exposure Research (BILDER), Department of Chemistry, North Carolina State University, Raleigh, NC 27695 USA

**Keywords:** IR-MALDESI, Quantitative mass spectrometry imaging, Electrospray doping, Ammonium fluoride, Glutathione

## Abstract

Increasing ion abundance in mass spectrometry is essential for enhancing detection, quantification, and understanding of biomolecules involved in key cellular processes. Infrared matrix-assisted laser desorption electrospray ionization (IR-MALDESI), MALDI, and nano-DESI have shown improved ion abundance when integrating specific concentrations of ammonium fluoride into the sample preparation and/or ionization steps. Herein, the importance of electronegativity and atomic size for the proposed mechanism of increased ion abundance is evaluated by testing a variety of ammonium halide salts. Ammonium fluoride was confirmed to result in the largest increase in ion abundance and was subsequently used to perform quantitative mass spectrometry imaging (qMSI) of glutathione (GSH) in healthy mouse liver tissue. Using ~70 µM NH_4_F as an ESI dopant, up to a ~ two-fold increase in ion abundance was observed for these biomolecules, as well as an improvement in the limit of detection, detection frequency, and quantification of endogenous GSH in tissue.

**Graphical Abstract:**

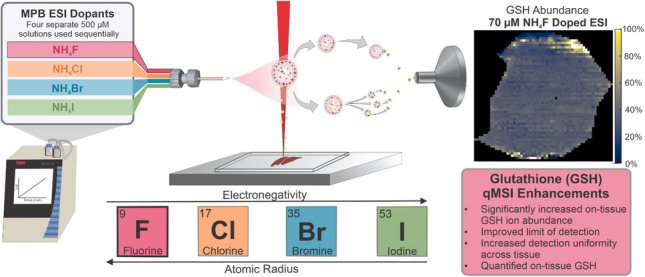

**Supplementary Information:**

The online version contains supplementary material available at 10.1007/s00216-025-06198-z.

## Introduction

Mass spectrometry imaging (MSI) is a specific application of mass spectrometry that allows for the detection and spatial localization of thousands of analytes within a biological sample to reveal 2D or 3D morphological details imperative for understanding cellular processes without the need for labeling [1, 2]. However, MSI abundances are qualitative in nature and cannot provide precise analyte concentrations in a sample, which is vital for tracking the degree to which biomolecules of interest are up- and down-regulated in wildtype versus disease models. Quantitative mass spectrometry imaging (qMSI) allows for the accurate determination of the absolute concentration of analytes within a biological sample [3], usually requiring internal standards. Using infrared matrix-assisted laser desorption electrospray ionization (IR-MALDESI), a hybrid ionization technique combining matrix-assisted laser desorption ionization (MALDI) and electrospray ionization (ESI), a wide array of biomolecules can be measured [4]. Previous experiments using IR-MALDESI have optimized qMSI using on-tissue calibration curve techniques [3] and investigated alternative electrospray dopants [5] and solvents [6] to enhance biomolecule detection and quantification accuracy. However, doping the standard electrospray solvent with ammonium salts, which at discreet concentrations has reportedly resulted in a significant impact on detected ions, abundance, and signal-to-noise ratio, has yet to be optimized for IR-MALDESI qMSI applications.

Other techniques, such as MALDI-MSI, nano-DESI-MSI, and LC-MS, have investigated ammonium halide electrospray and matrix additives to enhance biomolecule ion abundance. MALDI-MSI found a threefold increase [7] in lipid signal using 500 µM ammonium fluoride as a matrix additive, nano-DESI-MSI found a 10–110-fold [8] increase in signal across 4 lipid classes using 500 µM ammonium fluoride as an ESI dopant, and LC-MS found a 2–22-fold increase [9] in small molecule signal using 200 µM ammonium fluoride as an ESI dopant. Recently, the use of NH_4_F as an ESI dopant for IR-MALDESI was reported, where up to eightfold and fourfold increases in signals were observed for lipids and glycans at 70 µM and 350 µM NH_4_F ESI dopant, respectively [10].

Ammonium fluoride is proposed to increase abundance because the highly electronegative fluoride ions capture protons to form [M-H^+^]^−^ ions. This proposed mechanism can be further explored by studying the impact of other ammonium halide ESI dopants, such as ammonium chloride, ammonium bromide, and ammonium iodide. Beyond electronegativity, the location of these salts in the ESI droplets can play a role in their ability to interact with analytes, based on theoretical and computational models showing smaller, more electronegative halides reside on the interior of droplets, while larger, less electronegative halides reside at the air–water interface as a product of differing ion solvation energies [11, 12]. Additionally, more recent studies have pointed to differences in halide-water cluster structures where chloride, bromide, and iodide structures exhibit similar arrangements, while fluoride-water clusters display qualitatively different and more specific three-dimensional configurations because of stronger interactions [13]. To further explore this mechanism, the effects of ammonium fluoride, chloride, bromide, and iodide ESI dopants were compared using a lipid mixture, providing a greater understanding of the effect of increased electronegativity, acid-character, and decreased atomic radius. The effect of ammonium halide doping was also evaluated on glutathione (GSH), an essential metabolite and biomolecule of interest, for qMSI-based analyses. Glutathione plays crucial roles in treating chronic liver diseases such as nonalcoholic fatty liver disease (NAFLD) [14], regulating and detoxifying the cardiovascular system [15], and promoting tumor progression and chemotherapy resistance when upregulated [16]. Accurately quantifying glutathione and understanding how to improve its detection and on-tissue visualization are important steps to take to further our understanding of its metabolic capacity in healthy and disease states.

In this study, we demonstrate that utilizing ammonium fluoride as an ESI dopant for IR-MALDESI results in optimal negative mode abundance of lipids and GSH when compared to other ammonium salt ESI dopants. Ammonium fluoride, at its optimized concentration, increased these biomolecule signals up to ~ two-fold, improved the limit of detection, resulted in more uniform detection of analyte across tissue, and allowed for the quantification of endogenous GSH, matching previously reported values.

## Experimental

### Materials

Acetonitrile (ACN), acetic acid, LC-MS grade water, LC-MS grade methanol (MeOH), and 1-mm microscope glass were purchased from Thermo Fisher Scientific (Nazareth, PA, USA). A Hamilton 7000.5 syringe was purchased from Hamilton Company (Reno, NV, USA). Nitrogen gas was purchased from Arc3 gases (Raleigh, NC, USA). Glutathione (NAT-GSH), ammonium fluoride (> 98.0%), ammonium chloride (99.5%), ammonium bromide (> 99.0%), and ammonium iodide (99.5%) were purchased from Sigma Aldrich (St. Louis, MO, USA). Splash Lipidomix™ was purchased from Avanti Research (Alabaster, AL, USA). Homoglutathione (hGSH) was purchased from Bachem (Torrance, CA, USA), and stable isotope labeled glutathione (^13^C_2_ ^15^N–SIL-GSH) was purchased from Cambridge Isotopes Laboratories (Tewksbury, MA, USA).

### Sample preparation

A healthy, fresh frozen mouse liver was obtained from the Ghashghaei laboratory at North Carolina State University (NCSU) College of Veterinary Medicine where all animal experiments were in accordance with the NCSU Institutional Animal Care and Use Committee (IACUC, #19–811-B). The frozen liver was stored at −80 °C before sectioning to a thickness of 7 µm using a Leica CM1950 cryostat (Buffalo Grove, IL, USA) at −15 °C and thaw mounting to 1-mm thick glass slides [17].

For qMSI, prior to thaw mounting each liver section for analysis, glass slides were homogenously sprayed with a normalization standard of 1.00 mg mL^−1^ homoglutathione (hGSH) [18] in 50% methanol, using a pneumatic sprayer (TM Sprayer, HTX Imaging, Carrboro, NC, USA). Using the evenly coated slides, each liver section was thaw mounted, and a serial dilution series of ^13^C_2_^15^N (SIL-GSH) in 50:50 (v/v) methanol/water was prepared with the following concentrations: 0 (blank), 0.031, 0.062, 0.13, 0.25, 0.50, and 1.0 mg mL^−1^^19^. Immediately prior to IR-MALDESI qMSI analysis, liver sections were spotted with 100 nL of each SIL-GSH solution using a Hamilton 7000.5 syringe [19]. For this experiment, two and three technical replicates were collected for the non-ESI-doped and ESI-doped conditions, respectively.

### IR-MALDESI source

All well plate and tissue analyses were conducted using the Next Generation IR-MALDESI source coupled to an Orbitrap Exploris 240 Mass Spectrometer (Thermo Fisher Scientific, Bremen, Germany) [20]. Standard methods utilizing this IR-MALDESI source were employed for these analyses, as described previously [4]. To highlight important details for these experiments, each sample was positioned inside of a humidity-controlled enclosure on an XYZ-controlled Peltier-cooled stage. A mid-IR laser (JGM Associates, Inc., Burlington, MA, USA) at 2940 nm was fired at eight pulses per burst, with 2.0 mJ/burst reaching the sample to resonantly excite O–H stretching bands of endogenous water in aqueous solutions and tissues, as well as exogenous water in the ice matrix applied to tissue. These laser shots result in the desorption of neutral molecules from the sample that are intercepted by an orthogonal electrospray plume before being ionized in an electrospray ionization-like manner. In this case, the various ESI mobile phases were flowed at 1.2 µL/min using an EASY nano-LC pump (Thermo Fisher Scientific) and an applied voltage of 3.6 kV. Data were collected in negative, centroid mode with ions analyzed from *m/z* 150 to *m/z* 1000 and multi-injection ratio at 7. Resolving power was set to 240,000_FWHM_ at *m/z* 200 with EASY-IC internal calibrant fluoranthene ([M^●+^] *m/z* 202.0777), using an injection time of 15 ms. Additionally, automatic gain control was disabled, and the S-lens RF value was set to 70%.

### IR-MALDESI ammonium salt dopant exploration

To measure the ion abundance of Splashmix and GSH solutions, a well plate was positioned on the sample stage, and 30 µL of each solution was aliquoted directly prior to analysis. For the investigation of all ammonium salt ESI dopants, multiple mobile phases were made, with mobile phase A (MPA) being comprised of 50% ACN and 1 mM acetic acid [21] and mobile phase B (MPB) being comprised of 50% ACN, 1 mM acetic acid, and 500 µM of a specified ammonium salt. To determine both the optimal ammonium salt ESI dopant and concentration, the EASY nano-LC pump was programmed with a gradient from 5 to 95% MPB, corresponding to an ESI dopant concentration range of 25–475 µM.

Once the optimal dopant and concentration were determined, the optimized concentration of MPB was used for all future experiments. For imaging experiments, liver sections were thaw mounted onto a 1-mm glass slide and placed on the sample stage. To facilitate the formation of an ice matrix, the enclosure was purged with nitrogen gas to obtain < 12% relative humidity (RH) [22]. The stage was then cooled to −10 °C, held constant until thermal equilibrium was achieved, the nitrogen flow stopped, and the enclosure was opened to expose the stage to ambient humidity [22]. The increase in RH formed a thin layer of ice on top of the tissue; the enclosure was then subsequently closed and purged again with nitrogen to prevent excessive ice formation. At this point, the LC gradient method was started, and ablation was initiated in a meandering pattern with 150 × 150 µm circular laser spots.

### IR-MALDESI qMSI GSH analysis

For liver tissue GSH qMSI experiments, an intact mouse liver was serially sectioned where replicates 4, 5, 7, 11, and 12 underwent the sample preparation previously outlined. The Easy nano-LC was programmed to run continuously using the optimized dopant concentration, 70 µM NH_4_F. A diffractive optical element (DOE) was installed in the optical train of the IR-MALDESI source [23], which allowed for the ablation of 180 × 180 um square spots used to quantify the concentration of natural glutathione (NAT-GSH) in each liver section using a spatial calibration curve calculated by MSiReader v3.5 (MSI Software Solutions, Raleigh, NC) [24, 25].

### Data analysis

All raw mass spectra were analyzed using XCalibur (Thermo Fisher Scientific). These RAW files were then imported for analysis in MSiReader [26]. All ion images were produced with an *m/z* tolerance of ± 2.5 ppm.

For qMSI data evaluation, the absolute spatial calibration curve tool in MSiReader was used. An increased sample size (*n* = 30) [19] was used within each tissue, and data was extracted using square bioinformatic regions of interest (ROIs) to capture the average abundance of NAT-GSH for each ROI in equivalent sampling areas of 0.81 mm. The abundance of each voxel within the ROI was then exported; the average abundance across that area was calculated, and the obtained average abundance was used to calculate the concentration of NAT-GSH using an SIL-GSH spatial calibration curve output by MSiReader for each tissue.

## Results and discussion

### Effect of ammonium salt ESI dopants on aqueous lipid ion abundance

**Fig. 1:**
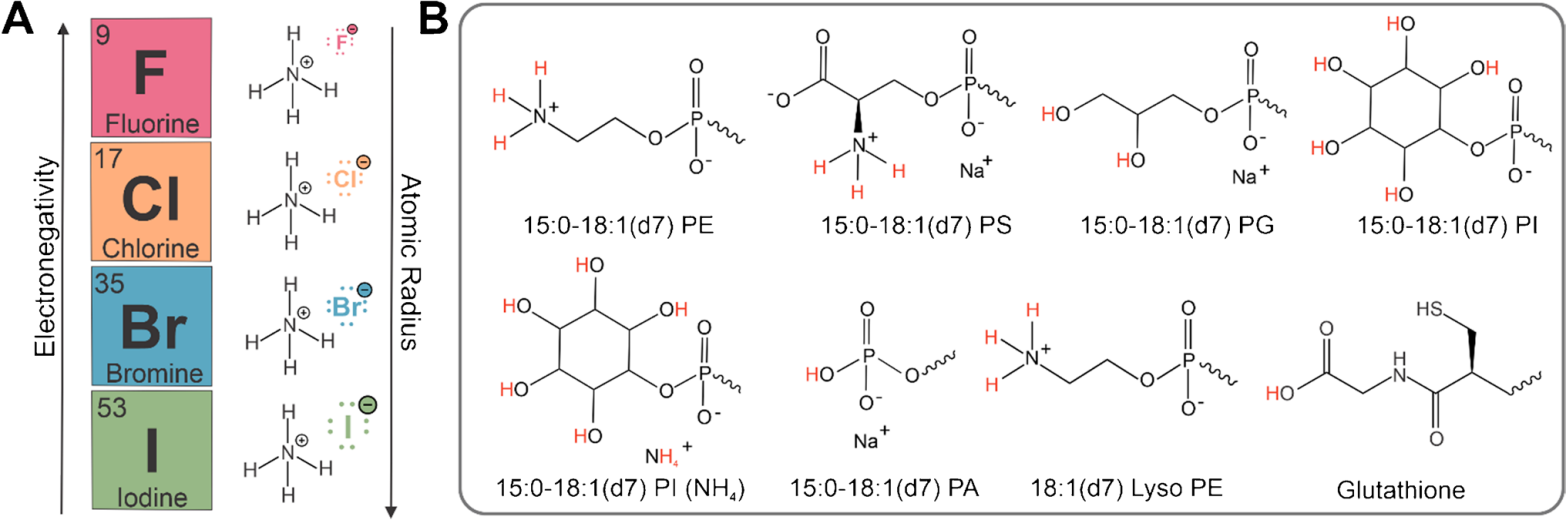
**A** Periodic trends that contribute to the behavior of the selected ammonium salt dopants illustrating the inverse trend of electronegativity and atomic radius. **B** Head group-focused deprotonation sites and theorized abstracted protons (in red) of selected lipids and glutathione

To investigate the hypothesized mechanism behind ammonium salt ESI doping in negative ion mode, ammonium fluoride, ammonium chloride, ammonium bromide, and ammonium iodide were selected as ammonium halides that have a range of differing electronegativities, acid-base behaviors, and atomic radii (Fig. 1A). This difference in electronegativity and size allowed for the extrapolation of periodic trend-influenced behavior of each dopant within the electrospray. Additionally, noting that fluoride ions have the weakest conjugate base character, followed by chloride, bromide, and iodide ions, the dissociation of each proton scavenger from the ammonium counterion can assist in understanding signal enhancement differences. Several glycerophospholipid subclasses were selected for this analysis, as well as the bioactive peptide and metabolite, glutathione (Fig. 1B).

**Fig. 2:**
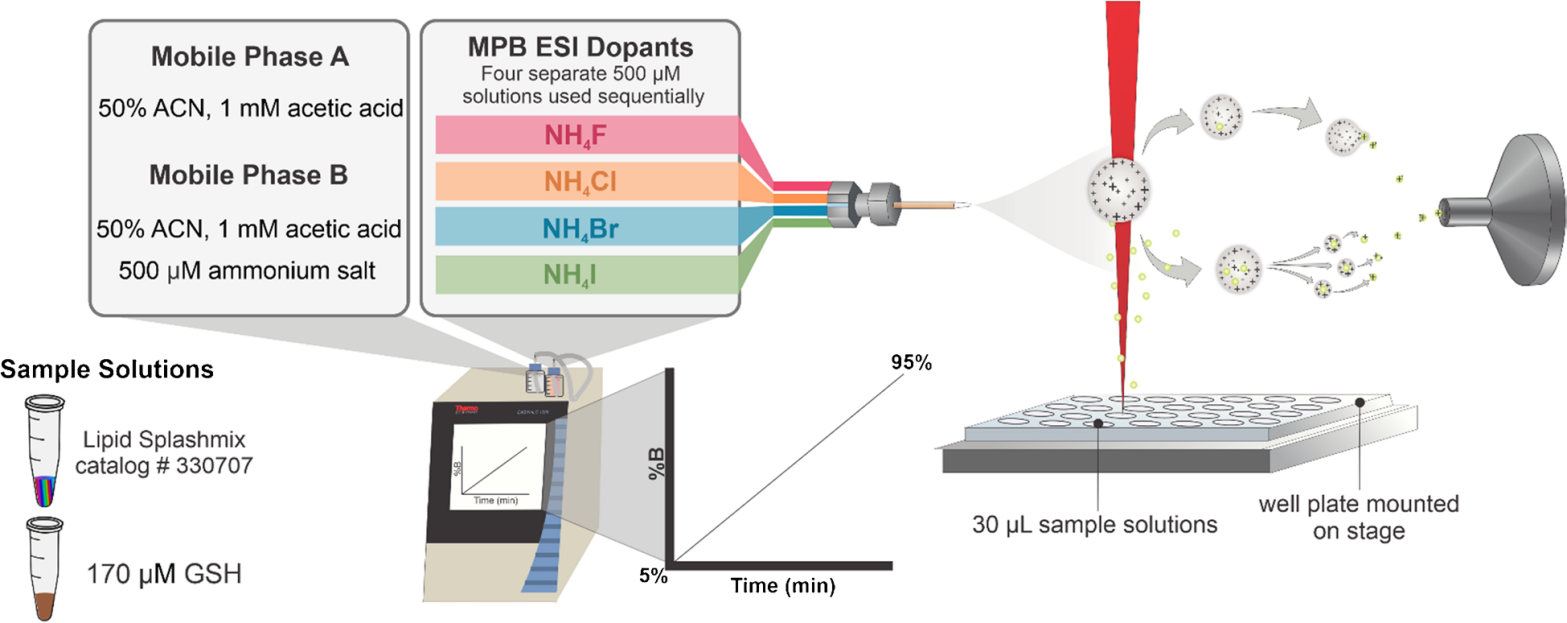
Experimental workflow for the determination of the optimal ammonium salt dopant and concentration for maximum ion abundance of aqueous sample solutions. An Easy-nano LC with MPA (50% ACN and 1 mM acetic acid) and MPB (50% ACN, 1 mM acetic acid, and 500 µM selected ammonium salt) was used with a continuous gradient increasing from 5 to 95% MPB, corresponding to ammonium salt concentration increase from 25 to 475 µM

To determine the optimal concentrations associated with each dopant for maximum ion abundance, NH_4_F, NH_4_Cl, NH_4_Br, and NH_4_I were used to analyze Splashmix and GSH solutions over a continuous gradient increasing from 25 to 475 µM (Fig. 2).

**Fig. 3:**
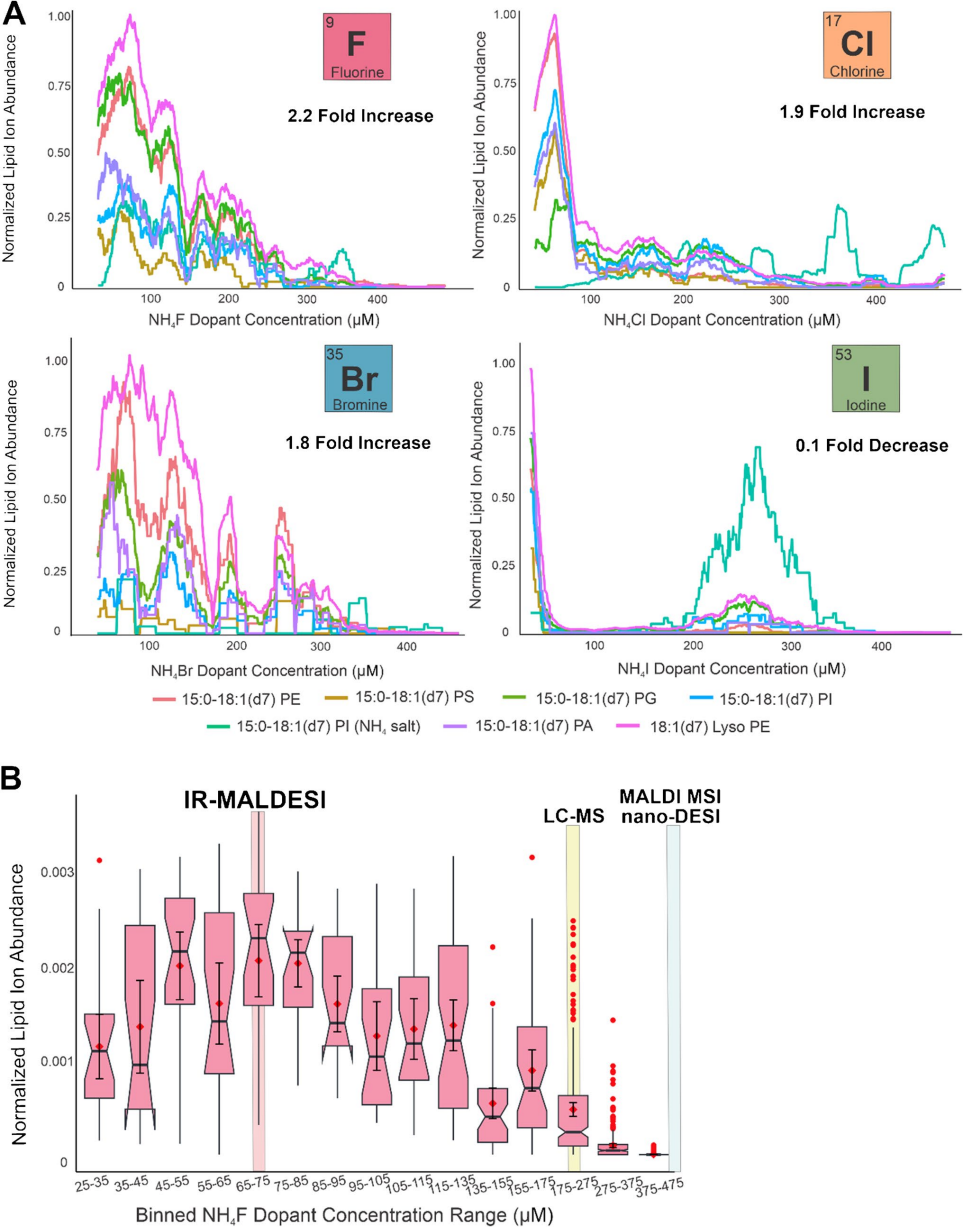
**A** Box-car averaged plots (window size = 50) of max-normalized lipid ion abundances utilizing NH_4_F reproduced from previous work for direct comparison between NH_4_Cl, NH_4_Br, and NH_4_I ESI dopants. The largest fold-change increase was observed for NH_4_F which was calculated using the dopant concentration corresponding to the maximum lipid ion abundance. **B** Notched boxplot depicting ~ 70 µM NH_4_F (median value for 65–75 µM bin), which was reproduced from previous work, as the optimal concentration for TIC-normalized maximum lipid ion abundance. Recent LC-MS, MALDI-MSI, and nano-DESI-MSI findings were also contrasted with these IR-MALDESI results

Over the course of the gradient, lipid ion abundances increased, achieving 2.2-fold, 1.9-fold, and 1.8-fold improvements once reaching ~ 70 µM NH_4_F^10^, ~ 50 µM NH_4_Cl, and ~ 70 µM NH_4_Br, respectively (Fig. 3A). This increase was observed across several subclasses of glycerophospholipids, including phosphatidylethanolamines (PE), phosphatidylserines (PS), phosphatidylglycerols (PG), phosphatidylinositols (PI), phosphatidic acids (PA), and lysophosphatidylethanolamines (LPE). Meanwhile, in the case of NH_4_I, all lipid ion abundances decreased to 10% of the original value immediately upon ESI doping, with a resurgence of partial signal at ~ 260 µM NH_4_I (Fig. 3A**)** [10]. These fold-change values accounted for the average of all lipid ion abundances at the two LC-gradient data points that correspond with the optimized dopant concentration. As each ESI dopant increased beyond the optimal concentration, in the cases of NH_4_F, NH_4_Cl, and NH_4_Br, increased ionization of background ions led to ion suppression and increased noise. In the case of NH_4_I, triiodide ions (MMA ± 4.47 ppm) formed a suppressive peak that quickly overcame all background and lipid ion detection. This suppression occurred until the signal fractionally and briefly returned for both background ions and lipid ions, most notably for the phosphatidylinositol NH_4_^+^ salt. These ammonium iodide findings are consistent with proposed theoretical and computational models which suggest that iodide resides on the exterior of water droplets [12]. In this case, it is postulated that strong surface and low gas-phase proton affinities cause iodide to act more as a charge carrier or component to form non-covalent adducts rather than as a proton scavenger in negative mode ionization. This lack of proton abstraction is further validated by evaluating the strong conjugate base character of iodide ions, which discourages disassociation from ammonium and leads to a large presence of intact NH_4_I in solution.

The final NH_4_F concentration, 70 µM, was more closely confirmed via a notched boxplot which accounted for the behavior of all measured lipids normalized to the total ion current [10] (Fig. 3B). Unlike previous reports, the optimal concentration found for IR-MALDESI was obtained using a continuous gradient to measure every concentration of dopant from 25 to 475 µM. This methodology allows for more global optimization, rather than the determination of a local maximum. Using IR-MALDESI as the ionization source resulted in a lower ammonium fluoride dopant concentration required for maximized lipid ion abundance in comparison to concentrations tested for nano-DESI and MALDI (Fig. 3B). However, there are differences in these technologies, and the previously reported concentrations may remain optimal for those techniques.

The application of this optimized ammonium fluoride dopant concentration for enhanced on-tissue lipid detection resulted in an average eight-fold increase of the aforementioned native lipid subclasses [10].

### Effect of ammonium salt ESI dopants on GSH ion abundance

The optimal ammonium salt and concentration for GSH were first determined by analyzing the ion abundance of a solution over the gradient (Fig. 2). The same trend as observed with Splashmix was observed for GSH with the use of ~ 70 µM NH_4_F, ~ 50 µM NH_4_Cl, ~ 70 µM NH_4_Br, and ~ 50 µM NH_4_I enhancing the signal 1.8-fold, 1.1-fold, 1.0-fold, and 0.2-fold, respectively. As salt electronegativity increases and size decreases, the ability of the ammonium salt to effectively abstract protons increases, resulting in [M-H]^−^ ion signal enhancement. This trend supports previously suggested theoretical models [12, 13] and further demonstrates that the higher solvation of fluoride, which results from its smaller radius and higher charge density, forces it to reside on the interior of negatively charged ESI droplets and implicates it as a better proton scavenger as it interacts with H^+^ more in “bulk.”

Once again, the optimized ~ 70 µM NH_4_F concentration was confirmed via a notched boxplot showing that the median of the 65–75 µM bin corresponded with the highest GSH ion abundance (Supplemental Fig. 1). Additionally, the 175–275 µM bin was statistically significantly different from the adjacent concentration bins, showing a local maximum peak of GSH ion abundance. This peak was directly compared against the bin from 65 to 75 µM using a Wilcoxon signed rank test to determine the statistical significance between medians, and the *P*-value 7.15 × 10^–7^ confirmed a statistically significant increase for the 65–75 µM bin. To visualize the impact that ~ 70 µM NH_4_F ESI doping had on signal enhancement of [M-H^+^]^−^ GSH ions (*m/z* 306.0765) and the isotopic peaks of GSH (*m/z* 307.0799 and *m/z* 308.0724), a raw mass spectra reflection plot was generated comparing a non-ESI-doped spectra to a ~ 70 µM NH_4_F ESI-doped spectra (Supplemental Fig. 1).

**Fig. 4:**
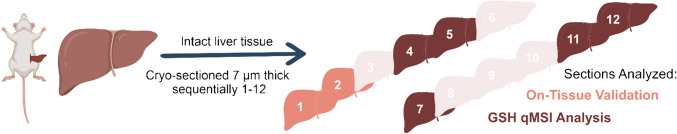
Liver tissue technical replicate details highlighting sections used for liver tissue (orange) and GSH qMSI analyses (brown), as well as those discarded (white). Sections 1 and 2 were used for liver tissue imaging, while sections 4, 5, 7, 11 and 12 were used for GSH qMSI analysis

To verify that increased sample complexity would not change the optimized NH_4_F ESI dopant concentration for GSH analysis, liver tissue sections were used for gradient analyses. Technical replicates of the liver sections used for this endogenous GSH validation and the subsequent GSH qMSI analyses are shown in Fig. 4.

**Fig. 5:**
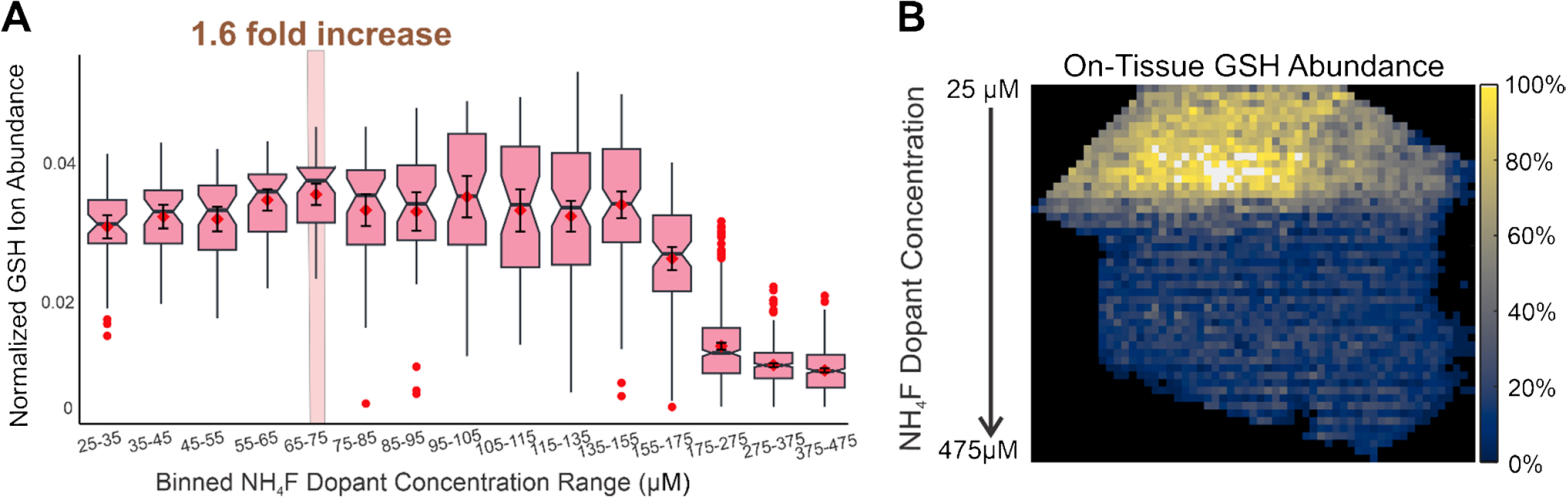
**A** Notched boxplot depicting ~ 70 µM NH_4_F (median value for 65–75 µM bin) as the optimal concentration for TIC-normalized maximum on-tissue GSH ion abundance. There was a 1.6-fold increase in on-tissue GSH ion abundance utilizing ~ 70 µM NH_4_F ESI dopant. **B** Corresponding liver tissue GSH heatmap depicting on-tissue NH_4_F concentration gradient data is consistent with results of the well-plate analysis where highest abundance appears close to the beginning of the gradient. One hundred percent corresponds to the maximum GSH ion abundance measured across tissue

Due to the relative homogeneity of liver tissue, a direct comparison can be made between the abundance of on-tissue ions and each NH_4_F concentration, allowing for a clear representation of the impact of NH_4_F ESI doping on GSH ion abundance (Fig. 5A and B). These results were consistent with the well-plate analysis, with the median of the 65–75 µM bin reporting the highest GSH ion abundance and confirming ~ 70 µM NH_4_F as the optimal ESI dopant concentration for maximized on-tissue GSH ion abundance.

### Effect of ammonium fluoride ESI dopant on qMSI of GSH

**Fig. 6:**
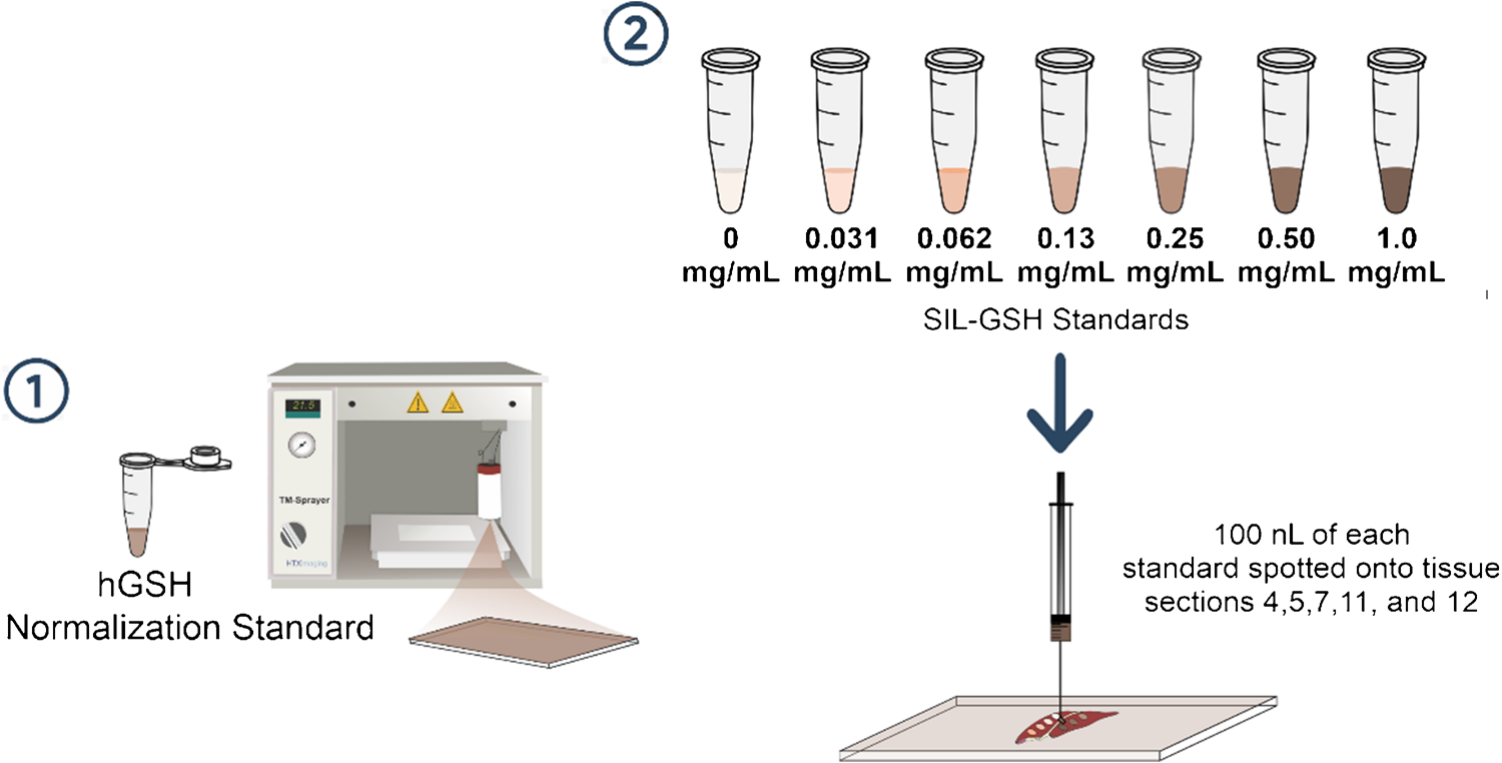
Sample preparation for GSH qMSI analysis of liver tissue sections. Homoglutathione was homogenously sprayed on all slides to serve as a normalization standard. A serial dilution series of SIL-GSH was prepared for each tissue, minimizing risk of contamination between spotting. Finally, 100 nL of each SIL-GSH standard was spotted onto each tissue directly prior to IR-MALDESI qMSI analysis

With an increase in on-tissue GSH ion abundance, quantitative mass spectrometry imaging experiments were conducted utilizing tissue samples thaw-mounted onto hGSH-coated glass slides, spotted with SIL-GSH, and analyzed with the ~ 70 µM NH_4_F ESI dopant (Fig. 6).

**Fig. 7:**
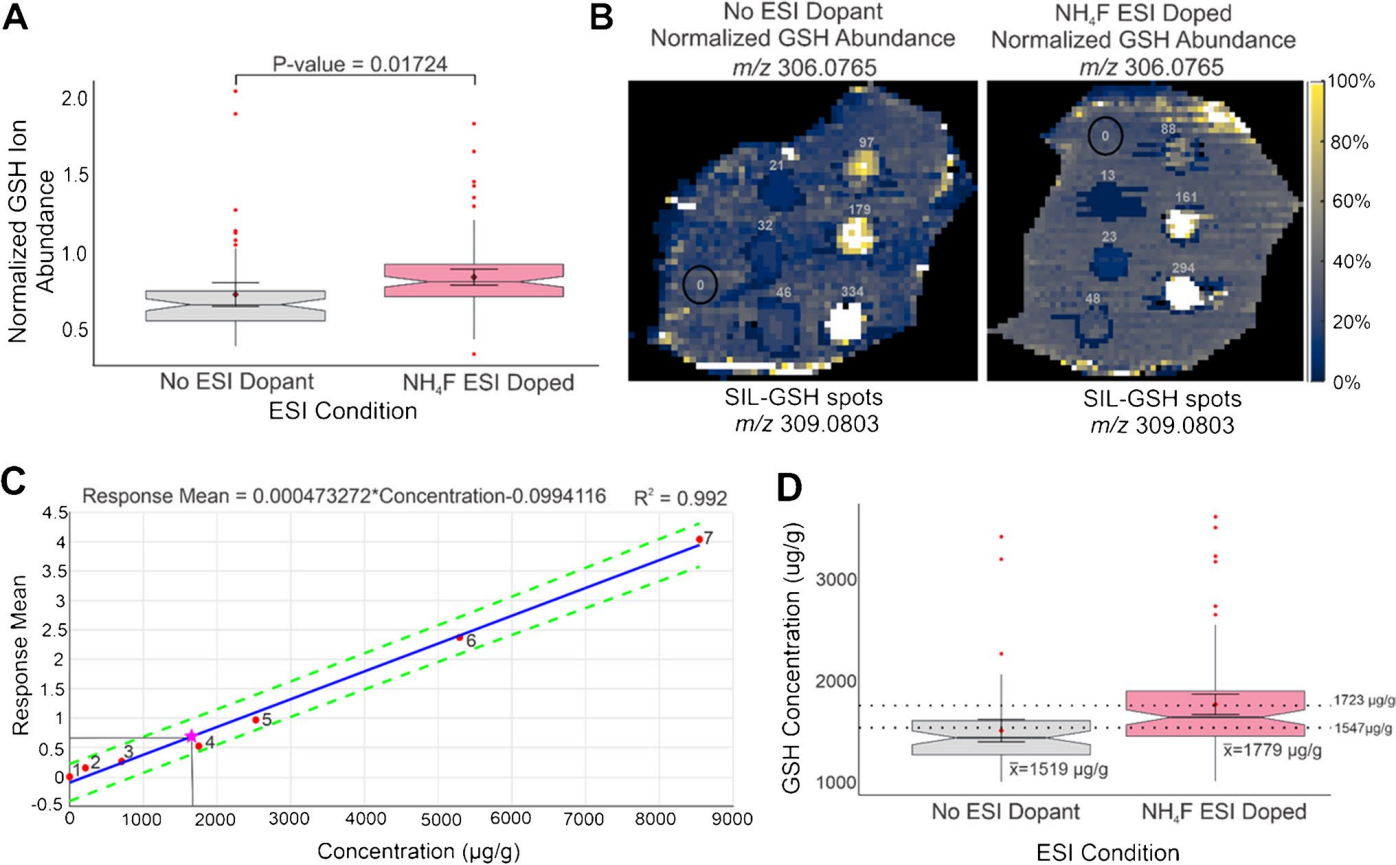
**A** Normalized GSH ion abundance for *N* = 3 technical NH_4_F ESI-doped replicates and *N* = 2 technical control replicates with 30 bioinformatic ROIs averaged per tissue, with a statistically significant difference indicated by *P*-value = 0.01724 between groups. **B** Representative GSH heatmaps of control versus ESI-doped tissues further reinforcing that NH_4_F doping results in increased GSH ion abundance, but also more uniform detection across tissue. SIL-GSH spots were overlaid for each tissue, and the molar amount of SIL-GSH applied per unit area was reported (in grey) in pmol/mm^2^ for each spot. **C** Representative SIL-GSH spatial calibration curve from ~ 70 µM NH_4_F ESI-doped tissue section 4 used to quantify the endogenous NAT-GSH concentration in tissue. **D** Notched boxplot revealing that the final, average on-tissue GSH concentration values for *N* = 2 and *N* = 3 technical replicates for non-doped ESI and NH_4_F ESI-doped tissues agree with a previously reported range suggesting that this ESI doping does not have a negative impact on quantification accuracy

Seventy micrometers of NH_4_F ESI doping resulted in a significant increase in GSH ion abundance across *N* = 3 technical replicates and 30 bioinformatics ROIs averaged per tissue, as determined by a Wilcoxon signed rank test (Fig. 7A). Raw, identically scaled, representative heatmaps of this NAT-GSH data, which also indicate the location and actual molar amount of SIL-GSH standard spots on tissue per unit area, were generated. The concentration of each SIL-GSH standard dispensed per unit area was reported in grey on each heatmap, and the black circle on each tissue represented the location of the 0 mg mL^−1^ SIL-GSH spot; the abundance within these two circles simply reflects NAT-GSH (Fig. 7B). For the non-ESI-doped tissue, 0 (blank), 21, 32, 46, 97, 179, and 334 pmol mm^−2^ of 0 (blank), 0.031, 0.062, 0.13, 0.25, 0.50, and 1.0 mg mL^−1^ SIL-GSH standards were spotted, respectively. In the same sequence, the NH_4_F-ESI-doped tissue had 0 (blank), 13, 23, 48, 88, 161, and 294 pmol mm^−2^ spotted. These heatmaps revealed that ~ 70 µM NH_4_F ESI dopant not only significantly improved GSH ion abundance for qMSI, and therefore the limit of detection, but also resulted in more uniform detection across the entirety of the tissue. This enhancement in ion abundance and limit of detection after ammonium fluoride ESI doping is imperative for providing more robust and accurate data for mouse liver tissue GSH concentration calculations.

The concentration of NAT-GSH was calculated for each tissue using the Absolute–QMSI spatial calibration curve tool in MSiReader to generate SIL-GSH calibration curves (Fig. 7C). The final on-tissue GSH concentrations calculated were 1519 µg/g tissue and 1779 µg/g tissue for non-doped and ~ 70 µM NH_4_F ESI-doped analyses, respectively (Fig. 7D). While the final values are different, the control and NH_4_F-doped qMSI results lie close to the 1547–1723 µg/g range previously reported for NAT-GSH in healthy mouse liver [19], revealing that ~ 70 µM NH_4_F ESI doping enhances on-tissue GSH ion abundances, improves limit of detection, and does not have a negative impact on quantification accuracy.

## Conclusions

This study has demonstrated that ammonium fluoride ESI doping, with an optimized concentration of ~ 70 µM NH_4_F, in negative mode IR-MALDESI results in significant signal enhancement of lipids and the essential metabolite, glutathione. Increased electronegativity, decreased atomic radius, weak conjugate base character, and location within the interior of ESI droplets allow fluoride ions to abstract protons from lipids and GSH significantly more than other ammonium halides, leading to an increase of [M-H]^−^ ions. The use of this ESI dopant caused a significant, up to ~ two-fold increase in biomolecule ion abundance, an improvement in the limit of detection, and more uniform detection of GSH across liver tissue, while quantifying on-tissue GSH.

## Supplementary Information

Below is the link to the electronic supplementary material.

Supplementary Material 1 (DOCX 271 KB)

## Data Availability

All data is available upon request.
