# Supplementary material for: Exploring ammonium salt doping to enhance ion abundance for quantitative mass spectrometry imaging
Source: Anal Bioanal Chem. 2025 Nov 4;417(30):7035–44. doi: 10.1007/s00216-025-06198-z (PMC12680730; doi:10.1007/s00216-025-06198-z)
Supplement: Supplementary file 1 — Supplementary Material 1 (DOCX 271 KB) [file 216_2025_6198_MOESM1_ESM.docx]

**Supporting Information**

**Exploring Ammonium Salt Doping to Enhance Ion Abundance for Quantitative Mass Spectrometry Imaging**

Alora R. Dunnavant, Seth M. Eisenberg, and *David C. Muddiman

*Biological Imaging Laboratory for Disease and Exposure Research (BILDER), Department of Chemistry, North Carolina State University, Raleigh, NC 27695*

**Submitted to:** *Analytical and Bioanalytical Chemistry*

**Submitted**: September 1, 2025

**Supplemental Material**: 1 Page / 1 Supplemental Figure

**Keywords**: IR-MALDESI; quantitative mass spectrometry imaging; electrospray doping; ammonium fluoride; glutathione

***Author for Correspondence**

David C. Muddiman, Ph.D.

FTMS Laboratory for Human Health Research

Department of Chemistry

North Carolina State University

Phone: 919-513-0084

Email: [dcmuddim@ncsu.edu](mailto:dcmuddim@ncsu.edu)


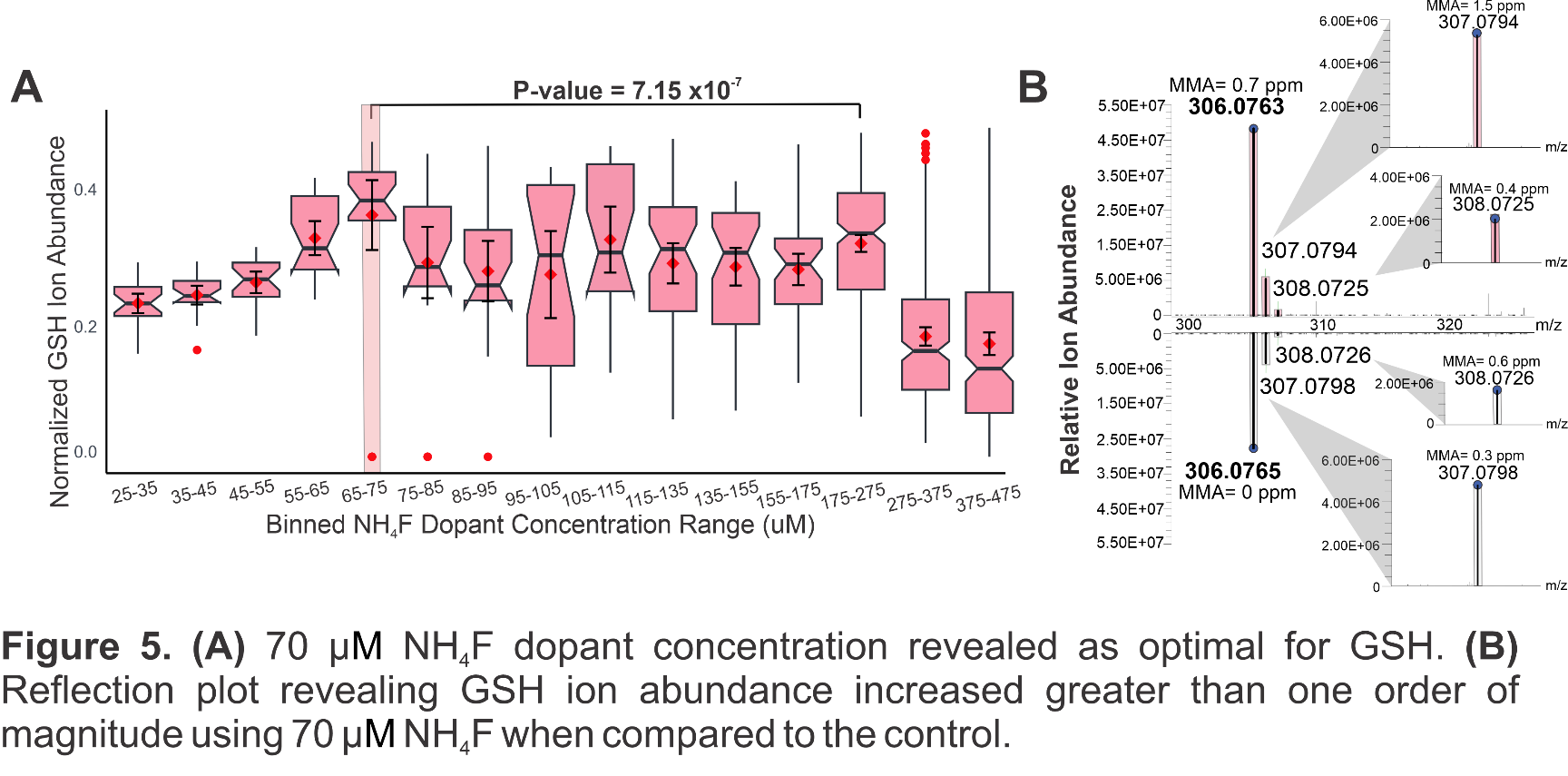


**Supplemental Figure 1**. **(A)** Notched boxplot depicting ~70 µM NH_4_F (median value for 65-75 µM bin) as the optimal concentration for maximum GSH ion abundance measured from a 170 µM GSH aqueous solution. A Wilcoxon Signed Rank Test used to calculate the p-value between the 65-75 µM and 175-275 µM bin medians revealed a statistically significant difference between them. This confirms ~ 70 µM NH_4_F ESI dopant concentration as optimal for GSH ion abundance enhancement. **(B)** Reflection plot of ~ 70 µM NH_4_F ESI doped spectrum for GSH and its isotopic peaks (top) versus the control, non-doped ESI spectrum for GSH (bottom). MMAs were also reported for each peak indicating high precision of the mass spectrometer.
